# Effect of gradually increasing force magnitude on the rate of canine retraction: a split mouth randomized controlled trial

**DOI:** 10.1186/s12903-026-08243-4

**Published:** 2026-04-21

**Authors:** Shady Abd El-Salam, Amira A. Aboalnaga, Wael A. Tawfik, Mona M. Salah Fayed

**Affiliations:** 1 Department of Orthodontics, Faculty of Dentistry, Cairo University, Cairo, Egypt; 2 Department of Orthodontics and Pedodontics, National Research Center, Cairo, Egypt

**Keywords:** Canine retraction, Force magnitude, Orthodontic tooth movement, Treatment duration, Gradual force

## Abstract

**Objective:**

To evaluate the effect of gradually increasing orthodontic force magnitude compared with a constant force magnitude on the rate of tooth movement during maxillary canine retraction.

**Methods:**

This split-mouth randomized controlled clinical trial included 15 patients (30 sides). Each patient received both interventions. On the intervention side, canine retraction was performed using nickel–titanium closed-coil springs delivering progressively increased forces of 50 g, 100 g, and 150 g over three consecutive monthly intervals. On the control side, a constant force of 150 g was applied throughout the study period. Maxillary arch impressions were obtained at baseline (T0) and at 4-week intervals for three months (T1, T2, and T3). Digital model analysis was used to assess the rate and total amount of canine movement.

**Results:**

No statistically significant difference was observed between the two sides in the monthly rate of canine retraction at any time point (*P* > 0.05). The total distance of canine movement was 2.58 ± 0.70 mm in the control side and 2.24 ± 0.82 mm in the intervention side, with no significant intergroup difference (mean difference = 0.34 ± 1.08 mm, *P* > 0.05).

**Conclusion:**

Gradually increasing orthodontic force magnitude did not enhance the rate of maxillary canine retraction compared with a conventional constant force protocol.

**Clinical relevance:**

Progressive force escalation during canine retraction does not provide a clinical advantage over constant force application, supporting the use of simpler, continuous force mechanics in routine orthodontic practice.

**Trial registration:**

The trial was registered in ClinicalTrials.gov Identifier: NCT05643443. https://clinicaltrials.gov/study/NCT05643443. Registered December 18, 2022.

**Supplementary Information:**

The online version contains supplementary material available at 10.1186/s12903-026-08243-4.

## Introduction

Comprehensive orthodontic treatment using fixed appliances is often associated with prolonged treatment duration, with an average length approaching 18–24 months [1, 2]. Extended treatment time may negatively influence patient motivation and compliance and has been linked to undesirable clinical sequelae such as enamel decalcification, periodontal inflammation, and external root resorption [3–5]. Consequently, reducing the overall duration of orthodontic therapy remains a persistent clinical objective.

Orthodontic tooth movement is fundamentally a biologic response to applied mechanical forces, resulting in a cascade of bone modeling and remodeling events within the periodontal ligament and surrounding alveolar bone. Compressive forces stimulate osteoclastic activity and bone resorption, whereas tensile forces promote osteoblastic bone formation. The rate and quality of tooth movement are influenced by multiple variables, including force magnitude, force continuity, tissue response, and individual biologic variability [6–8]. 

In recent years, considerable attention has been directed toward approaches aimed at accelerating orthodontic tooth movement. These strategies can be broadly categorized into surgical, pharmacological, and physical or mechanical modalities. Although surgically assisted techniques such as corticotomy-based procedures have demonstrated the potential to enhance tooth movement, their invasive nature, postoperative discomfort, and limited patient acceptance restrict their routine clinical application [9–14]. Pharmacological and physical adjuncts have also been investigated; however, inconsistent outcomes and limited clinical feasibility have prevented widespread adoption [15–18]. A recent systematic review summarized comparative outcomes of frictionless techniques (for example, T‑loops and closing loops) versus sliding mechanics and highlighted substantial heterogeneity in clinical effect sizes and outcomes across trials, indicating that mechanics alone do not fully explain differences in retraction efficiency [19]. 

From a biomechanical and biologic perspective, the magnitude and application pattern of orthodontic force play a pivotal role in determining the tissue response. Excessive forces may result in periodontal ligament hyalinization, delayed tooth movement, and increased patient discomfort, whereas lighter forces have been associated with more favorable biologic responses and efficient tooth displacement. Experimental and histological evidence suggests that gradually applied or light continuous forces may enhance osteoclastic recruitment while minimizing areas of hyalinization, potentially leading to more physiologic tooth movement with reduced adverse effects [20–23]. 

Despite this biologic rationale, limited clinical evidence exists regarding the effectiveness of gradually increasing orthodontic force magnitudes compared with conventional constant force application, particularly in the context of canine retraction. Furthermore, the impact of different force application strategies on anchorage preservation remains inadequately explored.

Therefore, the present randomized controlled clinical trial was designed to evaluate whether a progressively increased orthodontic force protocol influences the rate of maxillary canine retraction compared with a constant force regimen. In addition, the study aimed to assess the effect of both force systems on maxillary first molar anchorage during the retraction phase.

## Materials and methods

### Study design and ethical approval

This study was designed as a single-center, split-mouth, randomized controlled clinical trial conducted at the Orthodontic Department clinics, Faculty of Dentistry, Cairo University. Ethical approval was obtained from the institutional Research Ethics Committee. All participants received detailed explanations of the study procedures and provided written informed consent prior to enrollment. The trial was prospectively registered at ClinicalTrials.gov. Identifier: NCT05643443. This study was conducted and reported in accordance with the Consolidated Standards of Reporting Trials (CONSORT) guidelines [24]. A completed CONSORT checklist has been included as an additional file with this manuscript to ensure full transparency and reproducibility.

### Sample size calculation

The sample size was calculated based on the study by Aboalnaga et al. [9], which evaluated canine retraction rates under similar clinical conditions. That study reported a mean difference of 0.5 mm/month with a standard deviation of 0.6 mm. Accordingly, the standardized effect size (Cohen’s d) was estimated at 0.83.

Given the split-mouth design of the present study, a paired comparison model was assumed. Sample size estimation was performed using the General Linear Model (GLM) framework in SPSS (version 17.0; SPSS Inc., Chicago, IL, USA), with; Two-sided alpha level (α) = 0.05, power (1 − β) = 80%, three repeated measurements (M1, M2, M3), moderate within-subject correlation (*r* = 0.5).

Based on these assumptions, the minimum required sample size was 12 patients. To compensate for an anticipated dropout rate of approximately 20%, the sample size was increased to 15 patients (30 sides).

In this split-mouth design, the patient was considered the unit of randomization and the unit of analysis. Although each patient contributed two sides (control and intervention), these observations are nested within the same individual and are therefore not statistically independent. Accordingly, paired statistical methods were applied to account for within-subject correlation.

### Randomization and blinding

Random allocation of intervention sides was performed using computer-generated random sequences prepared by an independent individual (A.A). Allocation concealment was ensured using opaque sealed envelopes (M.S) prepared by a third party (M.S) who was not involved in the clinical procedure. Participants and outcome assessors were blinded to group allocation. However, operator blinding was not feasible due to the nature of the intervention.

### Participants and eligibility criteria

Patients were consecutively recruited from individuals seeking orthodontic treatment at Cairo University from January 2022 to January 2023. Eligible participants were males or females aged between 16 and 25 years, presenting with a full permanent dentition excluding third molars, and requiring bilateral extraction of the maxillary first premolars followed by canine retraction with moderate to maximum anchorage requirements. Only patients with satisfactory oral hygiene and healthy periodontal tissues were included.

Patients were excluded if they had systemic conditions, congenital or hereditary disorders, or were receiving medications known to influence bone metabolism or orthodontic tooth movement.

### Orthodontic preparation

Prior to the intervention phase, comprehensive clinical examination and diagnostic records were obtained for all participants. As part of a standardized preparatory phase, ready-made stainless steel bands^1^ were fitted and cemented on the maxillary first and second molars following separation. Fixed orthodontic appliances with a 0.022-inch Roth prescription^2^ were bonded^3^ to the maxillary teeth, excluding the incisors.

Posterior teeth were consolidated using stainless steel 0.012 ligature wire^4^. Within two weeks, extraction of the maxillary first premolars by a single operator (S.A.), after which leveling and alignment were initiated while bypassing the anterior segment. Archwire progression was individualized until a 0.017 × 0.025-inch stainless steel archwire^5^ was placed and maintained for one month prior to initiating canine retraction.

### Intervention protocol

Canine retraction was performed using nickel–titanium closed-coil springs^6^ attached between the maxillary canine and first molar hooks.

For the intervention side, Nickel–titanium closing coil springs were used to deliver gradually increasing forces: 50 g (1st month), 100 g (2nd month), and 150 g (3rd month). On the control side, Coil springs delivered a constant force of 150 g throughout the study period.

**Fig. 1:**
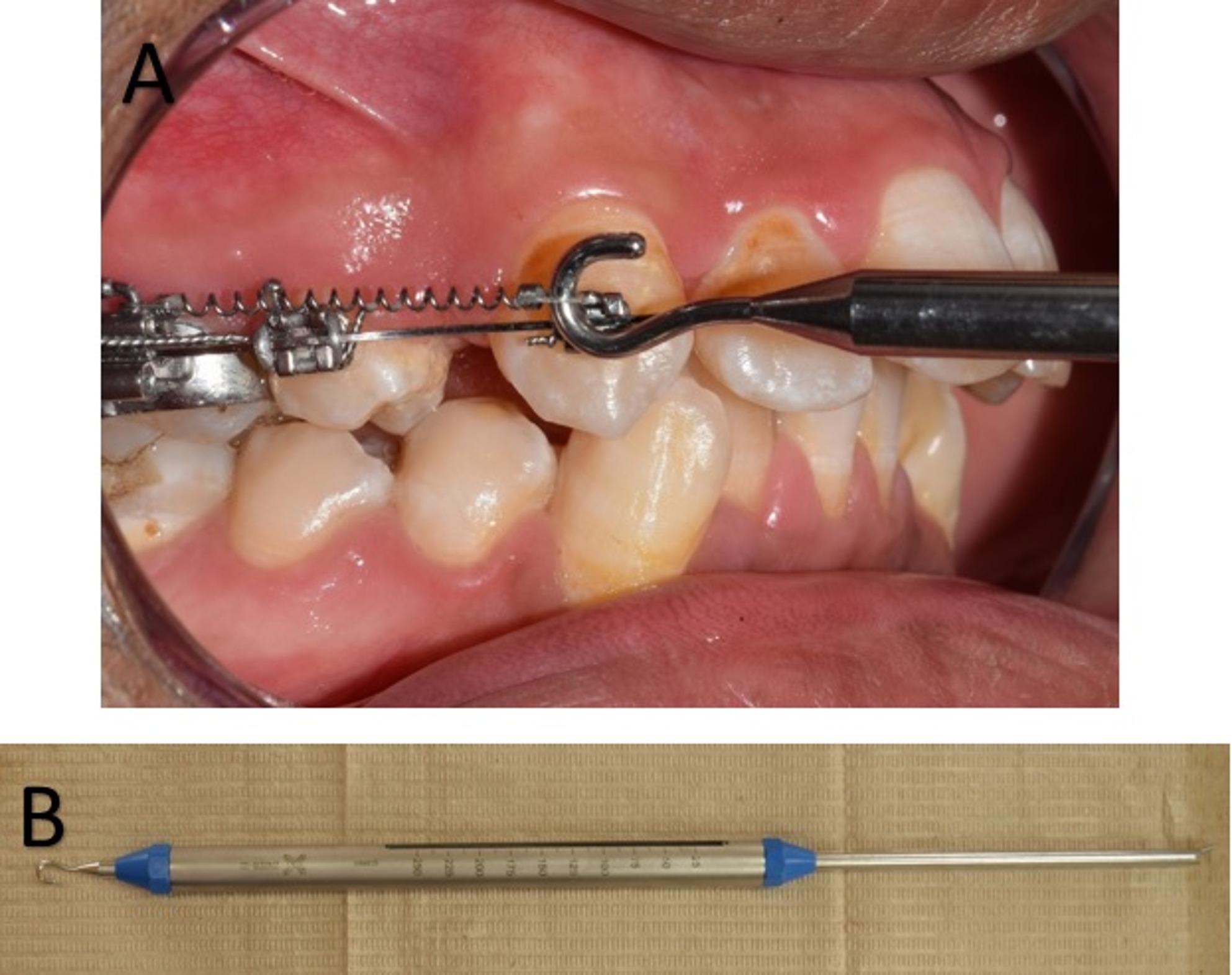
Morelli Force Gauge (A) used intra-orally to measure force level (B) Extra-oral view

Force levels were measured and calibrated intraorally at each visit using a Morelli orthodontic tension meter^7^ (Fig. 1). All measurements were performed by the same operator (S.A.) at the time of appliance placement and at each follow up visit. Force levels were verified every four weeks during routine clinical appointments. Follow-up visits were scheduled every four weeks for three months.

### Data collection

Maxillary impressions were obtained at four time points:

- T0: Baseline, before force application.
- T1: Four weeks after force application.
- T2: Eight weeks after the start of force application.
- T3: Twelve weeks after the start of force application.

Alginate impressions^8^ were poured in dental stone within 15 min to avoid dimensional distortion; then the stone models were trimmed, and labeled. The resulting stone models were then scanned using a Shining 3D DS-EX desktop dental scanner to generate STL files for digital analysis.

### Digital model analysis

**Fig. 2:**
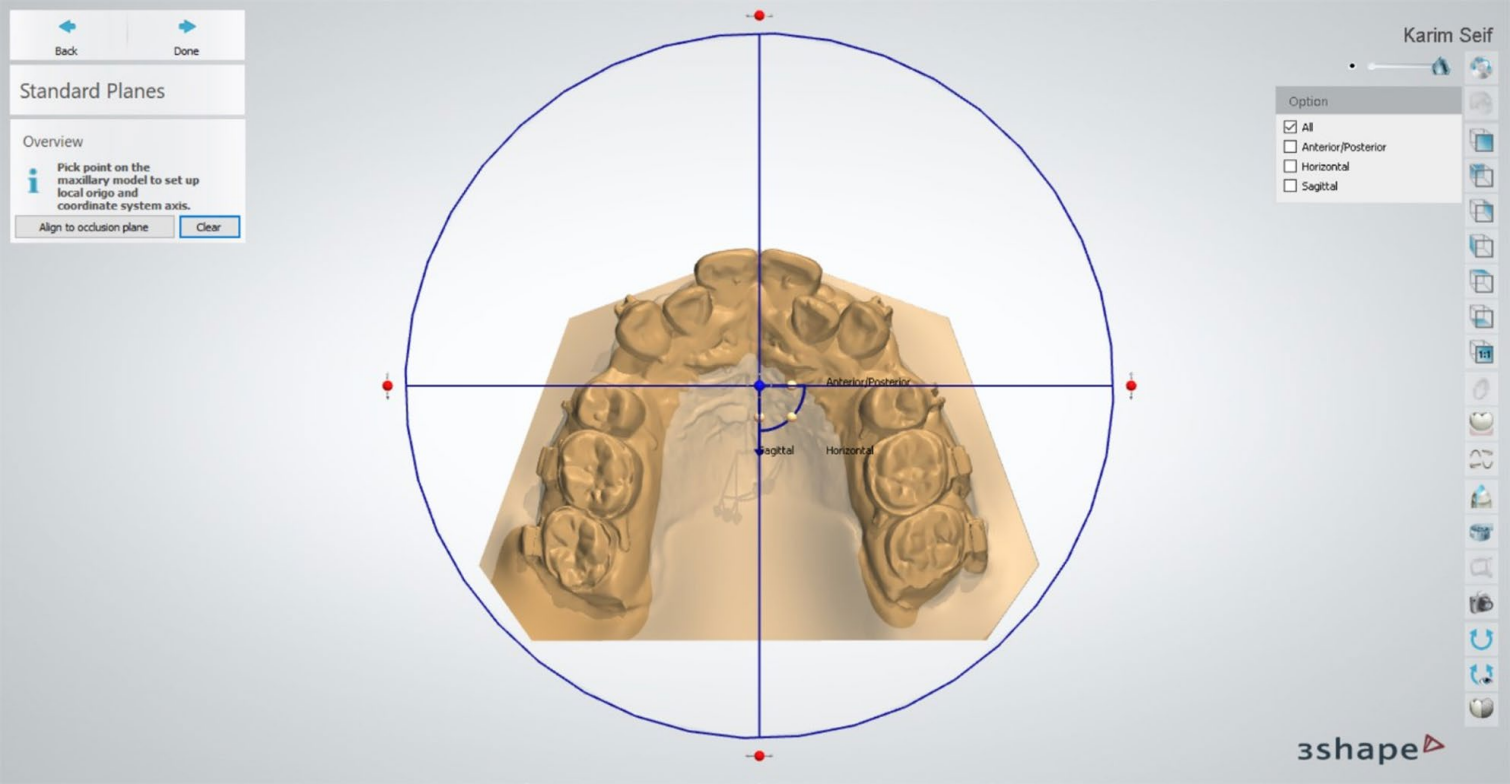
Orientation planes construction

3-Shape Analyzer software was used for superimposition and measurement:

Reference planes* (*Fig. 2*)*:

- *Occlusal plane*: Constructed from central fossae of maxillary first molars and contact point of maxillary central incisors.
- *Frontal plane*: Extended through three points at the third rugae, perpendicular to the occlusal plane.
- *Mid-sagittal plane*: Constructed using three mid-palatal points on the baseline model.

#### Superimposition

**Fig. 3:**
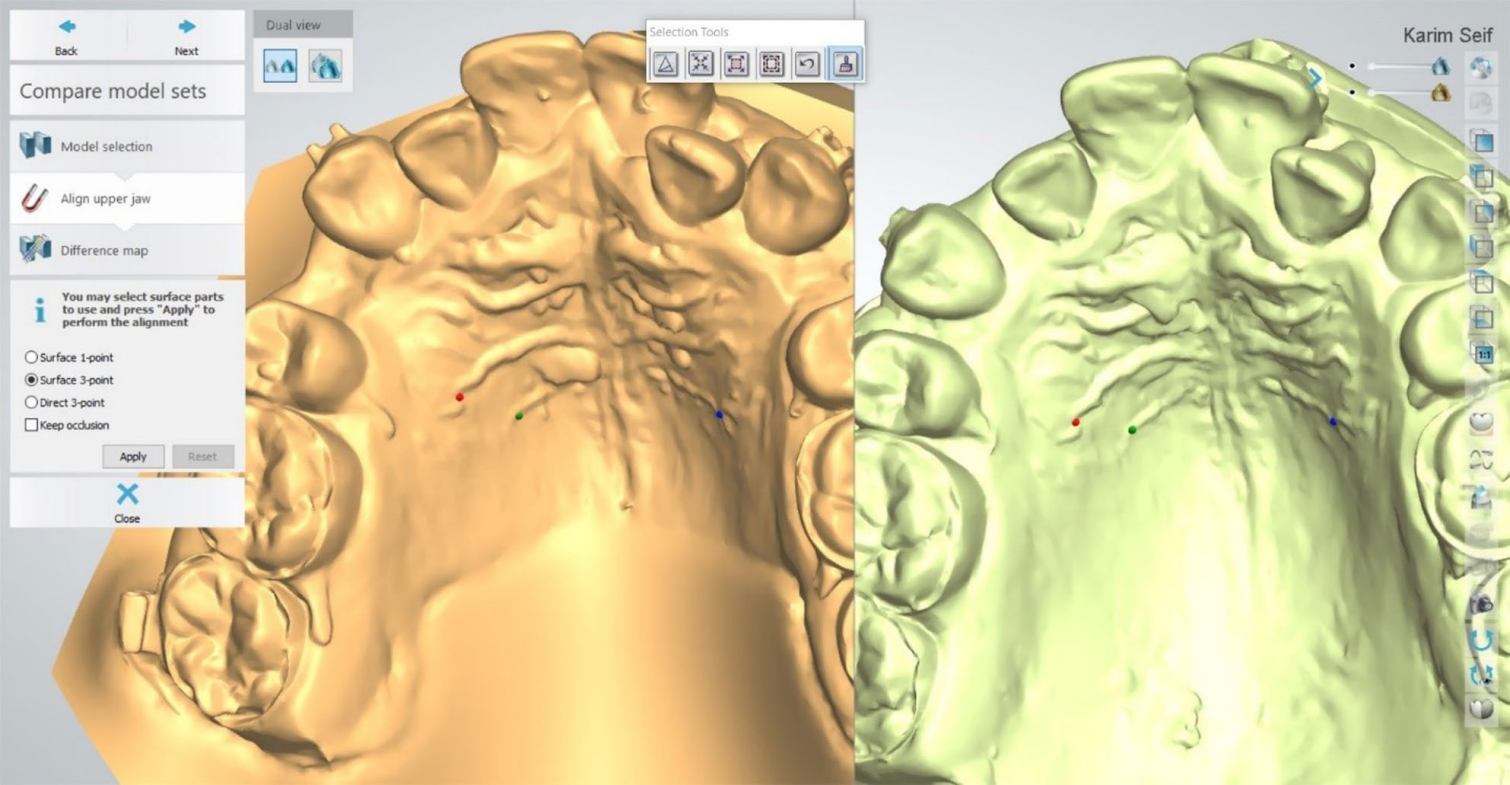
Surface three points superimposition (Points localization)

**Fig. 4:**
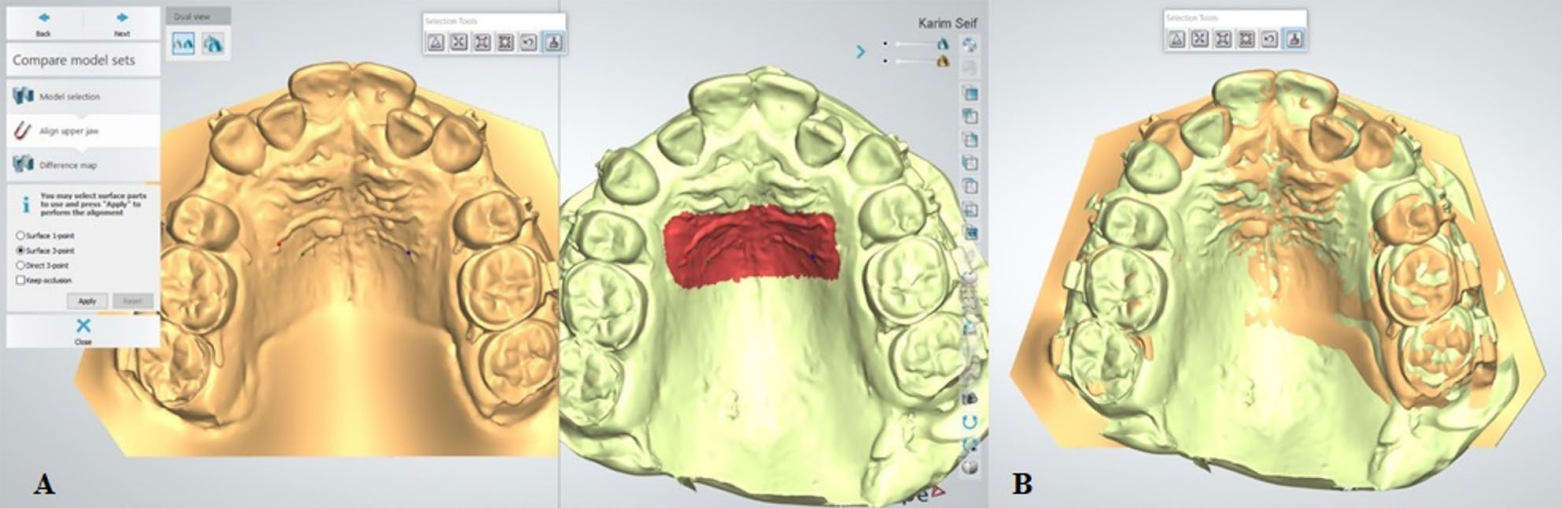
(**A**) Surface three points superimposition (Surface localization) (**B**) the final superimposed casts (T0 is “yellow” – T3 is “Green”)

Sequential models (T1–T3) were superimposed on T0 using a combination of 3-point surface registration (Fig. 3) and automated surface matching, with rugae as stable landmarks (Fig. 4A, B).

### Landmark identification (Fig. 5A):

- *Canine cusp tip*: Upper right (Rt CCT) and left (Lt CCT)
- *Mesiobuccal cusp of first molars*: Upper right (Rt MCT) and left (Lt MCT)

**Fig. 5:**
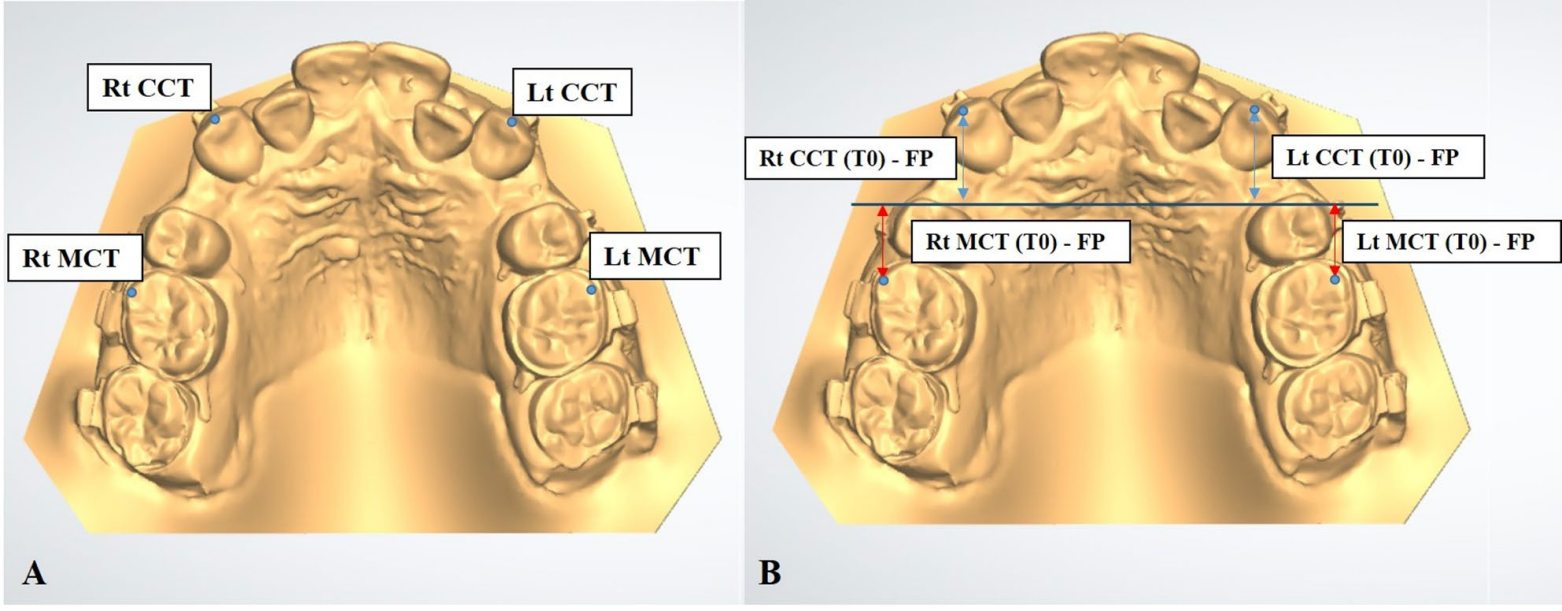
Landmarks identification & measurements representation (Rt CCT ; right canine cusp tip, Lt CCT ; left canine cusp tip, Rt MCT ; right first molar mesiobuccal cusp tip, Lt MCT ; left first molar mesiobuccal cusp tip, Rt CCT (T0) - FP ; the sagittal distance (mm) between the upper right canine cusp tip and the frontal plane (FP) in the digital model(T0), Lt CCT (T0) - FP ; the sagittal distance (mm) between the upper left canine cusp tip and the frontal plane (FP) in the digital model(T0), Rt MCT (T0) - FP ; the sagittal distance (mm) between the upper right first molar mesiobuccal cusp tip and the frontal plane (FP) in the digital model(T0), Lt MCT (T0) - FP ; the sagittal distance (mm) between the upper left first molar mesiobuccal cusp tip and the frontal plane (FP) in the digital model(T0))

Canine retraction was assessed by measuring the sagittal displacement of the canine cusp tip relative to the frontal plane at each time point (Fig. 5B). Monthly movement rates were calculated by determining the difference in canine position between consecutive models. Total canine displacement was calculated over the entire study duration.

Anchorage loss was evaluated by measuring the sagittal displacement of the mesiobuccal cusp tip of the maxillary first molar between baseline and the final follow-up model (Fig. 5B).

Landmark identification and measurements were performed independently by two blinded assessors, and mean values were used for statistical analysis. Measurements included the sagittal distance from the frontal plane to canine cusp tips (for retraction) and to first molar mesiobuccal cusp tips (for anchorage loss).

All digital measurements were performed by a calibrated principal assessor (S.A.). To assess inter-observer reliability, 20% of the models were randomly selected and independently remeasured by a second blinded examiner (A.A.). Inter-observer agreement was evaluated using Intraclass Correlation Coefficient (ICC) analysis based on a two-way mixed-effects model with absolute agreement. ICC values greater than 0.75 were considered indicative of good reliability while values above 0.90 were considered excellent reliability.

### Primary and secondary outcome measures

The primary outcome was the rate of canine retraction over successive monthly intervals (M1, M2, M3) and the total distance moved. The secondary outcome was maxillary first molar anchorage loss.

### Statistical analysis

Data analysis was performed using SPSS software (version 17.0; SPSS Inc., Chicago, IL, USA). Quantitative variables were summarized as mean ± standard deviation (SD) and 95% confidence intervals. Normality of distribution was assessed using the Shapiro–Wilk test.

Given the split-mouth design, paired samples t-tests were used to compare canine retraction and anchorage loss between the control and intervention sides at each time point. To evaluate the effects of side (control vs. intervention), time (T1, T2, T3), and their interaction on the rate of canine retraction, a two-way repeated measures ANOVA within the General Linear Model (GLM) framework was performed. Statistical significance was set at *P* < 0.05.

## Results

**Fig. 6:**
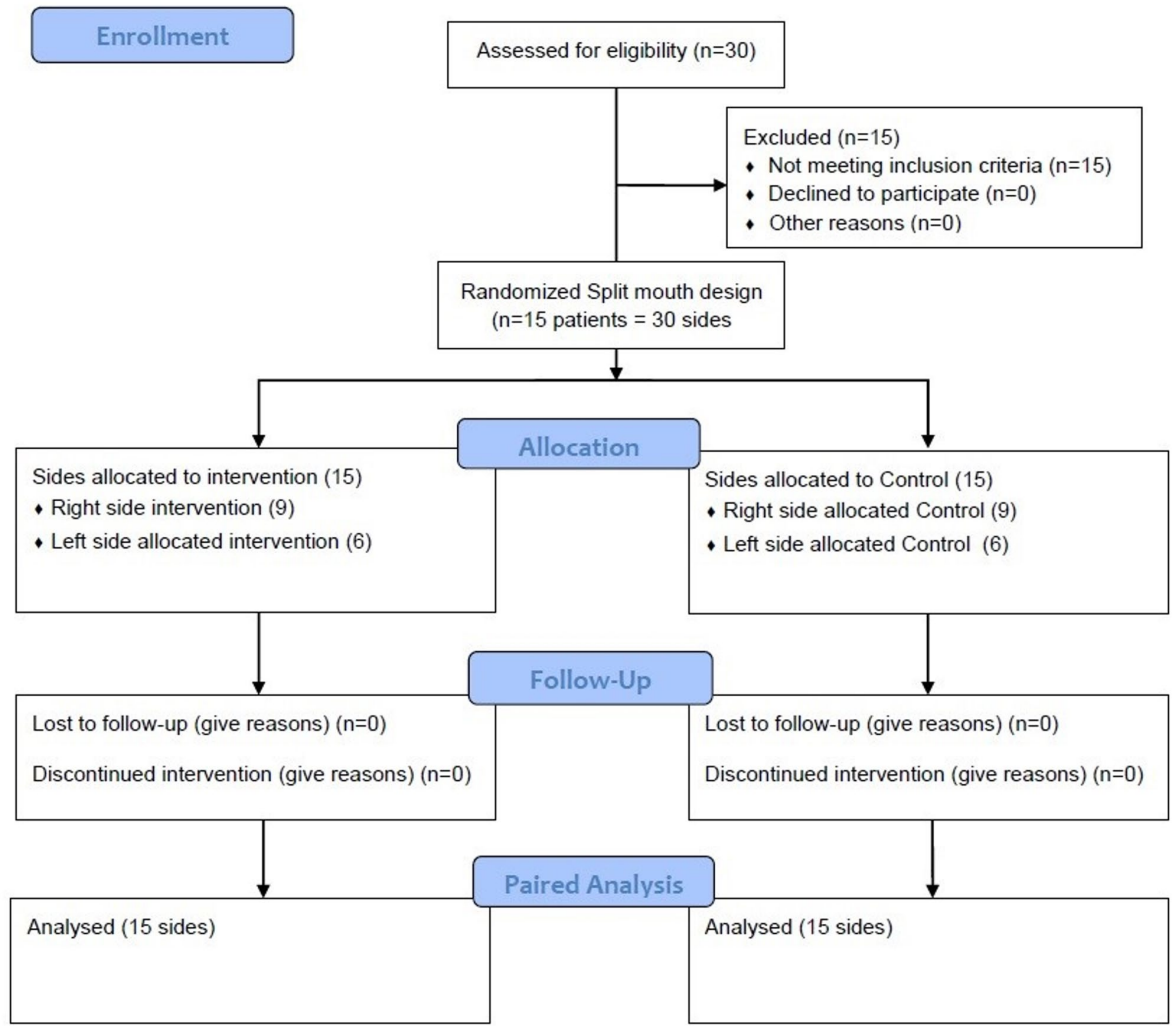
CONSORT flow chart showing patient flow during the trial

Of 30 patients assessed for eligibility, 15 were excluded for not meeting inclusion criteria (Bad oral hygiene: 6 patients; one or more previously extracted tooth: 6 patients; not in age range: 3 patients). 15 patients were randomized in a split‑mouth design (15 patients = 30 sides). All randomized patients completed the study and were included in the analysis (15 patients, 30 sides) (Fig. 6).

A total of 15 patients were randomized in this split-mouth design (15 patients = 30 sides). All randomized patients completed the study and were included in the analysis. The mean age in our study was 20.5 ± 3.85 years. Baseline demographic and clinical characteristics are presented in Tables 1 and 2. Because of the split-mouth design, age, sex, and malocclusion class were identical for both study sides and are therefore presented at the patient level (Table 1.A). Initial canine position at T0 was comparable between control and intervention sides (Table 2.B).

Table 3 summarizes the descriptive statistics for the rate and total distance of canine retraction in both the control and intervention sides across three monthly follow-up intervals (M1, M2, M3).

**Fig. 7:**
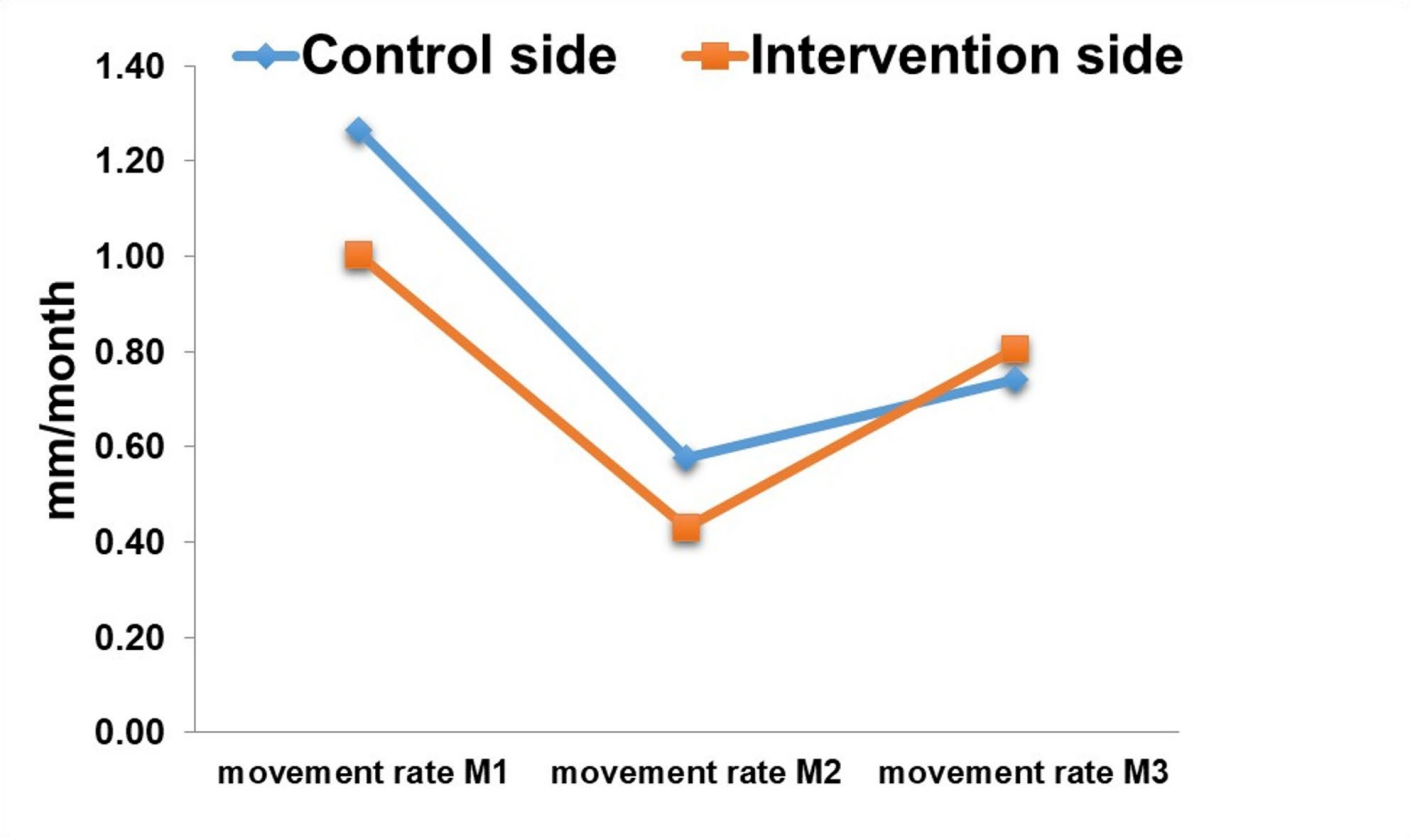
A line chart representing the rate of canine retraction between the control and intervention groupsover 3 months period

**Fig. 8:**
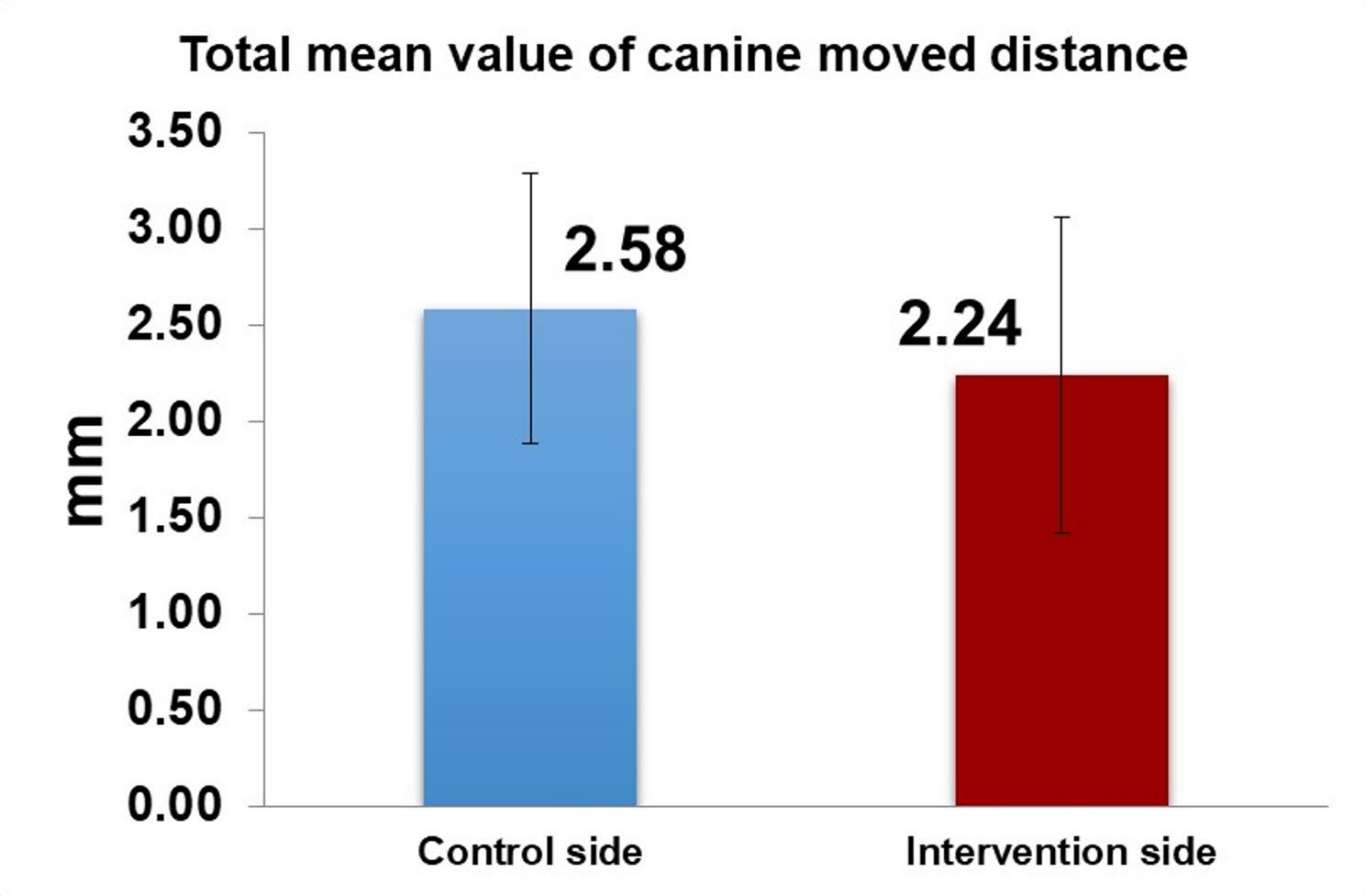
A bar chart represnting the total distance moved by the canines in the two groups in 3 months period

Paired samples t-tests showed no statistically significant differences in the rate of canine movement between the control and intervention sides at any individual time point (Fig. 7). The mean difference in the overall rate of canine movement was 0.11 ± 0.36 (*P*-value = 0.240). Similarly, the total canine displacement over the entire study period did not differ significantly between the two sides (Mean difference 0.34 ± 1.08 mm, *P*-value = 0.240) (Table 3) (Fig. 8).

A two-way repeated measures ANOVA with Greenhouse–Geisser correction revealed a significant main effect of time on the rate of canine retraction, F(1.58, 22.09) = 13.89, *p* < 0.001, partial η² = 0.50, 95% CI [0.21. 067], indicating that the rate of movement changed significantly across follow-up intervals. There was no significant main effect of side, F(1, 14) = 1.50, *p* = 0.240, partial η² = 0.10. The side × time interaction was also not statistically significant, F = 0.67, *p* = 0.508, partial η² = 0.05, suggesting that both sides followed a similar pattern of change over time. (Table 4)

Inter-examiner reliability was assessed using Intraclass Correlation Coefficient (ICC) analysis. The ICC values for canine retraction and anchorage loss measurements were 0.991 (95% CI: 0.96–0.99; *P* < 0.001), indicating excellent reliability.

### First molar anchorage loss

Table 5 presents the comparison of maxillary first molar anchorage loss between the two sides; which revealed insignificant difference (Mean difference = 0.17 ± 0.88, *P*-value = 0.477).

### Harms

No serious harm was observed throughout the study; routine checks didn’t reveal clinically meaningful force decay.

**Table 1:** Patient-level baseline characteristics of the study sample (*n* =15 patients)

Characteristic		Value
Age (mean ± SD)		20.5 ± 3.85 years
Sex n(%)		4 males, 11 females
Malocclusion class	Class I	4 (26.6%)
	Class II	11 (73.3%)

**Table 2:** Side-specific baseline characteristic (*n* = 15 control sides; *n* = 15 intervention sides) of pretreatment canine position

	Control side	Intervention side	Mean Difference	*P*-value
Initial canine position at T0 (mean ± SD)	11.82	12.11	-0.286 [-1.275-0.703]	0.545

**Table 3:** Statistics of Canine retraction rate & total Canine moved distance in control side vs. Intervention side for 3 months follow up period

Time	Mean		Mean difference	*P*-value
	Control Side *N* = 15)	Intervention Side (*N* = 15)		
M1	1.26 ± 0.61 (0.93–1.60)	1.00 ± 0.61 (0.66–1.34)	0.26 ± 0.80	0.226
M2	0.58 ± 0.37 (0.37–0.78)	0.43 ± 0.30 (0.26–0.60)	0.15 ± 0.63	0.385
M3	0.74 ± 0.45 (0.49–0.99)	0.81 ± 0.50 (0.53–1.09)	0.06 ± 0.75	0.747
Canine Total moved distance	2.58 ± 0.70 (2.19–2.97)	2.24 ± 0.82 (1.79–2.70)	0.34 ± 1.08	0.240
Mean rate of canine movement	0.86 ± 0.23 (0.73–0.99)	0.75 ± 0.27 (0.60–0.90)	0.11 ± 0.36	0.240

Independent sample t-test was used. Results are reported as Mean ± SD & 95% Confidence Interval below for each measurement

*M* month, *N* sample number, *C* Control side, *I* Intervention side, *SD* Standard deviation

*P*-value of < 0.05 is considered significant

**Table 4:** Two-Way Repeated Measures ANOVA Summary: Canine Retraction Rate

Source of Variation	Patial η²	df	F	*P*-value
Side	0.10 (0.00, 0.43)	(1, 14)	1.50	0.240
Time	0.50 (0.21, 0.67)	(1.58, 22.09)	13.89	< 0.001
Side × Time	0.05 (0.00, 0.22)	(1.84, 25.76)	0.67	0.508

A two-way repeated measures ANOVA was conducted. Sphericity correction method: Greenhouse–Geisser (GG), Partial η² is reported with two sided 95% CI

*P*-value of < 0.05 is considered significant

**Table 5:** First molar anchorage loss

First Molar Anchorage loss	Control side (*N* = 15)	Intervention side (*N* = 15)	Mean difference	*P*-value
	1.05 ± 0.67	0.88 ± 0.55	0.17 ± 0.88 (-0.32-0.66)	0.477

Paired samples t-test comparing 1st molar anchorage loss between control and intervention sides was conducted

*N* Number of patients, *SD* Standard deviation, *SEM* Standard error of the mean

*P*-value of < 0.05 is considered significant

## Discussion

The current study has tackled one of the historic shortcomings of orthodontic treatment, which is its lengthy duration. An average orthodontic treatment would usually last about 2 years, which could be very discouraging and unsatisfactory for our patients [25]. Prolonged treatment duration not only increases the risk of gingival irritation, decalcification, dental caries and root resorption [26], but also, and perhaps most critically, can exhaust patient cooperation, which further delays our treatment.

From the orthodontist’s point of view, brief orthodontic procedures enable more precise cost forecasting and the possibility to treat more patients [25]. As a result, it is an intriguing study issue to investigate ways to increase the movement and shorten the length of orthodontic therapy for both sake clinicians and their patients.

Using the attached 3 shape computer software (3-Shape Analyzer), the sequential digital models of each patient (T0-T3) were superimposed. Automated surface superimposition along with surface 3-point superimposition was done for more accurate superimposing. The points were selected on the right and left third rugae which were previously confirmed as stable palatal landmarks according to Shukla D et al. and van der Linden [27, 28]. This reliable method was followed in many former studies [9, 16, 29–32].

The effect of gradually increasing force magnitude on the rate of canine retraction did not accelerate tooth movement, as demonstrated by the findings in Table 3; Fig. 8. The mean difference in retraction rate between the control and intervention sides was 0.11 ± 0.36 mm, and the mean difference in total canine movement was 0.34 ± 1.08 mm (Table 3). Our finding that progressive force escalation did not increase canine retraction rate aligns with clinical trials showing limited or inconsistent acceleration from adjunctive interventions; for example, surgical micro‑osteoperforation produced modest, variable effects in split‑mouth randomized trials [9, 33–36].

Recent systematic reviews have highlighted that multiple adjunctive approaches aimed at accelerating orthodontic tooth movement, including surgical procedures, pharmacologic agents, and mechanical stimulation techniques, have produced variable and sometimes inconsistent clinical outcomes. These findings suggest that biological and biomechanical responses to orthodontic forces are multifactorial and not solely dependent on the application of higher force magnitudes. Consequently, the present findings support the concept that modifying force magnitude alone may not necessarily result in clinically meaningful acceleration of canine retraction [14, 19, 37]. 

We investigated the overall effect of time on canine retraction for the intervention and control sides. The two-way repeated measures ANOVA revealed no significant side × time interaction (*P* = 0.508) and no significant main effect of side (*P* = 0.204). This indicates that although the rate of retraction varied significantly over time (*P* < 0.001), the magnitude of change was similar between the two sides. These findings suggest that gradually increasing force magnitude did not produce a clinically or statistically significant enhancement in the rate of canine retraction, consistent with findings reported in previous clinical trials [9]. 

The observed peak in movement during the first month may be explained by an initial tipping phase followed by a period of up righting, as described by Andrews [38], which often results in reduced movement in later phases. With respect to total retraction distance, no statistically significant difference was observed between the sides. 

Anchorage loss was assessed by measuring the distance moved at the mesiobuccal cusp tip of the 1st molar as detected from the digital models. There was no statistically significant difference between both sides. Anchorage loss was clinically insignificant within each group as recorded (1.05 ± 0.67 mm for the control side and 0.88 ± 0.55 mm for the intervention side). Moreover, the included cases in this study did not require absolute anchorage. Although insignificant, the anchorage in the intervention side was less by around 17% than that in the control side (Table 5).

From the above-mentioned results, canine retraction by 50 gm of force rate of orthodontic tooth movement as 150 gm of force. Moreover, increasing the force to 100 gm did not result in any increase in the rate of orthodontic tooth movement. Luppanapornlarp S et al. confirmed that the rate of orthodontic tooth movement was corresponding between 50 gm vs. 150 gm of force in a two-month period [39]. Consequently it was concluded that canine retraction with 50 gm of continuous force magnitude proved to be efficient.

### Limitations

Several limitations should be acknowledged. First, the study evaluated canine retraction over only three months; longer-term effects on total treatment duration and post-treatment stability remain unknown. Second, the sample size (*n* = 15 patients) may limit detection of subtle differences. Third, the split-mouth design, while reducing inter-patient variability, may not fully eliminate cross-arch effects. Fourth, operator blinding was not feasible, which could introduce procedural bias, though outcome assessment was blinded. Fifth, only NiTi coil springs were used, and results may not generalize to other force delivery systems or bracket prescriptions. Finally, absolute anchorage was not required for included cases; findings may differ in patients requiring strict anchorage control.

### Clinical Relevance

Gradually increasing orthodontic force magnitude does not significantly accelerate canine retraction compared to continuous force. Light continuous forces (e.g., 50 g) are sufficient for efficient maxillary canine retraction, achieving comparable movement with minimal anchorage loss. Utilizing lighter forces may reduce periodontal stress and hyalinization, potentially improving patient comfort without compromising treatment efficiency.These findings support evidence-based optimization of force magnitude in routine clinical orthodontic practice, enabling effective tooth movement while minimizing adverse effects.

## Supplementary Information

Supplementary Material 1.

Supplementary Material 2.

Supplementary Material 3.

Supplementary Material 4.

## Data Availability

All the datasets used and analyzed are available from the corresponding author upon request.

## Footnotes

1 Washbon^®^. Orthodontic Bands, Ormco Supplies.

2 AO Mini Master Series.

3 BracePaste^®^ Adhesive, AO.

4 AO.

5 AO.

6 Ortho Organizer 0.010 × 0.030 9 mm NITANIUM COIL SPRING.

7 Force gauge (Morelli).

8 Hydrogum, Zhermack, Italy.

## Abbreviations

ANOVA: Analysis of Variance
ICC: Intraclass Correlation Coefficient
NiTi: Nickel–Titanium GLM: General Linear Model
CONSORT: Consolidated Standards of Reporting Trials
Gm: Gram of force
mm: millimeter
