# Supplementary material for: Effect of gradually increasing force magnitude on the rate of canine retraction: a split mouth randomized controlled trial
Source: BMC Oral Health. 2026 Apr 21;26:756. doi: 10.1186/s12903-026-08243-4 (PMC13126744; doi:10.1186/s12903-026-08243-4)
Supplement: Supplementary file 1 — Supplementary Material 1. [file 12903_2026_8243_MOESM1_ESM.docx]

**(Table 1): Landmarks & Measurements abbreviations and definitions**

| **Landmark abbreviation** | **Landmark definition** |  |
| --- | --- | --- |
| **Rt CCT** | The upper right canine cusp tip. |  |
| **Lt CCT** | The upper left canine cusp tip. |  |
| **Rt MCT** | The mesiobuccal cusp tip of the upper right 1st molar. |  |
| **Lt MCT** | The mesiobuccal cusp tip of the upper left 1st molar. |  |
| **Measurement abbreviation** | **Measurement definition** | |
| **Rt CCT (T0) - FP**  **Rt CCT (T1) - FP**  **Rt CCT (T2) - FP**  **Rt CCT (T3) - FP** | The sagittal distance (mm) between the upper right canine cusp tip and the frontal plane (FP) in digital model (T0), digital model (T1), digital model (T2) and digital model (T3) respectively. | |
| **Lt CCT (T0) - FP**  **Lt CCT (T1) - FP**  **Lt CCT (T2) - FP**  **Lt CCT (T3) - FP** | The sagittal distance (mm) between the upper left canine cusp tip and the frontal plane (FP) in digital model (T0), digital model (T1), digital model (T2) and digital model (T3) respectively. | |
| **Rt MCT (T0) - FP**  **Rt MCT (T3) - FP** | The sagittal distance (mm) between the mesiobuccal cusp tip of the upper right 1st molar and the frontal plane (FP) in digital model (T0) and digital model (T3) respectively. | |
| **Lt MCT - FP**  **Lt MCT - FP** | The sagittal distance (mm) between the mesiobuccal cusp tip of the upper left 1st molar and the frontal plane (FP) in digital model (T0) and digital model (T3) respectively. | |
