# Supplementary material for: Effect of gradually increasing force magnitude on the rate of canine retraction: a split mouth randomized controlled trial
Source: BMC Oral Health. 2026 Apr 21;26:756. doi: 10.1186/s12903-026-08243-4 (PMC13126744; doi:10.1186/s12903-026-08243-4)
Supplement: Supplementary file 2 — Supplementary Material 2. [file 12903_2026_8243_MOESM2_ESM.docx]

(Table 2) Statistics of Canine retraction rate & total Canine moved distance in control group vs Intervention group for 3 months follow up period

| Time | Mean | | Mean difference | P-value |
| --- | --- | --- | --- | --- |
|  | **Control Group**  **(N=15)** | **Intervention Group**  **(N=15)** |  |  |
| M1 | **1.26 ±**0.61  **(**0.93-1.60) | **1.00 ±** 0.61  (0.66- 1.34) | **0.26 ± 0.80** | **0.226** |
| M2 | **0.58 ±** 0.37  **(**0.37- 0.78) | **0.43 ±** 0.30  (0.26- 0.60) | **0.15 ± 0.63** | **0.385** |
| M3 | **0.74 ±** 0.45  (0.49-0.99) | **0.81 ±** 0.50  (0.53- 1.09) | **0.06 ± 0.75** | **0.747** |
| Canine Total moved distance | **2.58 ±** 0.70  (2.19- 2.97) | **2.24 ±** 0.82  (1.79- 2.70) | **0.34 ± 1.08** | **0.240** |
| Mean rate of canine movement | **0.86 ±** 0.23  **(**0.73- 0.99) | **0.75 ±** 0.27  (0.60- 0.90) | **0.11 ± 0.36** | **0.240** |

M; month, N; sample number, C; Control group, I; Intervention group, SD; Standard deviation

Results are reported as Mean ± SD & 95% Confidence Interval below for each measurement
