# Supplementary material for: Effect of gradually increasing force magnitude on the rate of canine retraction: a split mouth randomized controlled trial
Source: BMC Oral Health. 2026 Apr 21;26:756. doi: 10.1186/s12903-026-08243-4 (PMC13126744; doi:10.1186/s12903-026-08243-4)
Supplement: Supplementary file 3 — Supplementary Material 3. [file 12903_2026_8243_MOESM3_ESM.docx]

(Table 3) Two-Way Repeated Measures ANOVA Summary: Canine Retraction Rate

| Source of Variation | Sum of Squares | F | p-value |
| --- | --- | --- | --- |
| Group | **0.216** | **0.90** | **0.345** |
| Time | **6.444** | **13.44** | **<0.001** |
| Group × Time | **0.395** | **0.82** | **0.442** |

P-value of <0.001 is considered significant
