# Supplementary material for: Effect of gradually increasing force magnitude on the rate of canine retraction: a split mouth randomized controlled trial
Source: BMC Oral Health. 2026 Apr 21;26:756. doi: 10.1186/s12903-026-08243-4 (PMC13126744; doi:10.1186/s12903-026-08243-4)
Supplement: Supplementary file 4 — Supplementary Material 4. [file 12903_2026_8243_MOESM4_ESM.docx]

| First Molar Anchorage loss | Control group  (N=15) | Intervention group  (N=15) | Mean difference | P-value |
| --- | --- | --- | --- | --- |
| 1.05 ± 0.67 | 0.88 ± 0.55 | 0.17 ± 0.88  (-0.32-0.66) | 0.477 |  |

(Table 4) Paired samples t-test comparing 1st molar anchorage loss between control and intervention groups

N: Number of patients, SD: Standard deviation, SEM: Standard error of the mean.
